# Perspective: Controlling Epidermal Terminal Differentiation with Transcriptional Bursting and RNA Bodies

**DOI:** 10.3390/jdb8040029

**Published:** 2020-12-04

**Authors:** Duncan Wotherspoon, Clare Rogerson, Ryan F.L. O’Shaughnessy

**Affiliations:** 1 Centre for Cell Biology and Cutaneous Research, Blizard Institute, Queen Mary University of London, London E1 2AT, UK; d.j.wotherspoon@qmul.ac.uk; 2 The Francis Crick Institute, London NW1 1AT, UK; clare.rogerson@crick.ac.uk

**Keywords:** epidermis, terminal differentiation, transcriptional bursting, ribonuclear particles

## Abstract

The outer layer of the skin, the epidermis, is the principal barrier to the external environment: post-mitotic cells terminally differentiate to form a tough outer cornified layer of enucleate and flattened cells that confer the majority of skin barrier function. Nuclear degradation is required for correct cornified envelope formation. This process requires mRNA translation during the process of nuclear destruction. In this review and perspective, we address the biology of transcriptional bursting and the formation of ribonuclear particles in model organisms including mammals, and then examine the evidence that these phenomena occur as part of epidermal terminal differentiation.

## 1. Epidermal Terminal Differentiation Requires Increased Transcription during a Process of Nuclear Degradation

**Figure 1:**
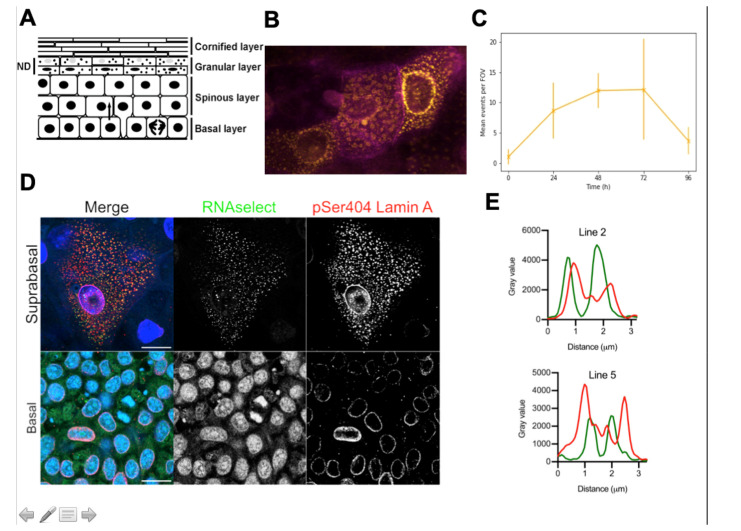
Evidence for Lamin A-positive ribonuclear protein granules in terminally differentiating rat epidermal keratinocytes. (**A**) Schematic of epidermal differentiation. Epidermal differentiation involves nuclear degradation (ND) and the loss of intracellular organelles (adapted from Rogerson et al., 2018). (**B**) pSer404 Lamin A expression (yellow) in the differentiating keratinocytes expressing keratin 10 (magenta) is observed in spherical cytoplasmic bodies [4,5]. (**C**) Expression of these bodies increases over time in confluent culture, peaking at 72 h post-confluency. (**D**) RNAselect detects cytoplasmic RNA bodies in suprabasal keratinocytes. Bar 20 µm (**E**). Profile plots across these bodies shows co-localisation of pSer404 Lamin A and RNA.

The principal function of the outer layer of the skin, the epidermis, is to provide a barrier to the external environment. The majority of this barrier is formed from dead but functional cells called corneocytes. In order for keratinocytes in the basal layer of the epidermis to differentiate into corneocytes, they undergo wide-ranging morphological and biochemical changes. They become rigid, water sealed and resilient by undergoing a developmental program which involves expressing large amounts of structural proteins as well as an array of enzymes which are used to covalently cross-link components of the cornified envelope (CE) [1,2]. The CE is the macromolecular structure that surrounds the corneocytes and forms the main barrier between the skin and the external environment. This process occurs concurrently with the complete destruction of all membrane bound organelles, including the nucleus (Figure 1A). Given that the hard copy of the genetic information is being destroyed, how can keratinocytes ensure that enough mRNA transcripts are present for polysomes to manufacture the protein needed to complete terminal differentiation?

Changes in gene expression observed in keratinocytes during terminal differentiation are orchestrated by a massive upsurge in transcriptional and translational activity. This is demonstrated by gene expression studies using immunofluorescence and in situ hybridisation for single genes, such as with the change in keratin pairs being expressed in differentiating epithelium [3]. We have determined that, consistent with this, in cultured rodent keratinocytes the onset of terminal differentiation is followed by an upsurge in transcriptional activity. Additionally, cytoplasmic phospho-lamin A-positive granules in keratinocytes are observed in terminally differentiated cells (Figure 1B,C) and are co-incident with cytoplasmic RNA (Figure 1D,E). Are these ribonucleoprotein (RNP) granules linked to the upsurge in transcriptional activity and if so, are they a sink for mRNA molecules whilst the nucleus is being destroyed?

Keratohyalin granules are electron-dense bodies observed in the upper layers of the epidermis. For structures recognised by pathologists for over 100 years, the function and formation of these bodies are remarkably unclear. Loss of these bodies is frequently associated with impaired epidermal terminal differentiation. Could they be RNP granules as well as the main region of filaggrin localisation in the epidermis [6,7] and possibly have other functions important to epidermal terminal differentiation? In this review, we assess the current literature on RNP granules and transcriptional bursting in developmental processes and discuss literature implicating the link between the two phenomena. We put particular emphasis on these processes in mammalian systems and cells that also destroy their nucleus, such as cells of the lens. We then apply this to the developmental and cell biology data in keratinocyte/epidermal literature to hypothesise potential functions that these phenomena may have in the process of keratinocyte terminal differentiation.

## 2. What Is Transcriptional Bursting—A Directed Process or just Noise?

One of the big mysteries of biology is why cells within a seemingly homogenous population have very heterogenous gene expression. Studies in the late-1990s and mid-2000s [8,9] showed that stochastic expression of reporter genes occurred in their respective cells, suggesting that transcription was discontinuous. This ‘transcriptional bursting’, with periods of inactivity and high activity, occurs in unicellular organisms such as the social amoeba *Dictyostelium discoideum* [10], *E. coli* [11], and in in vitro experiments in mammalian cells [12], demonstrating it to be an apparently ubiquitous phenomenon.

Transcriptional bursting behaviour is mainly determined by the probabilistic nature of the random events required for gene expression to occur, and the low numbers of molecules that are involved [13,14]. For transcription to occur, a series of sequential events must happen, such as the unfolding of chromatin to make promoters accessible to the basal transcription machinery and host of other molecules such as transcription factors. Once these complexes are assembled, their interaction with the DNA keeps the gene active. Therefore, the properties of the components of these complexes interacting which each other and with their cognate binding sequences will influence their kinetic behaviour, which influences their transcriptional behaviour [15,16,17,18,19,20]. Post-transcriptionally, the natural turnover of mRNA and proteins in a given cell will also influence the temporal effect of transcription [21]. These various factors result in ‘gene noise’: transcriptional events that are stochastic not deterministic [22].

In single-celled organisms, stochastic cell fate decisions allow for variability in the population, providing greater heterogeneity for cell survival despite the cells being genetically identical. This has been observed in bacteria, where a small minority of the population can enter a transient state where they can take up DNA under stress [23,24]. These cells had been exposed to exactly the same environment, meaning this state is independent of environmental and genetic cues.

The control of developmental transition in metazoans also appears to be controlled by pulsatile gene expression [25,26]. Noise likely plays a regulatory role in a wide range of cellular processes [27]. Gene expression noise results in some genes being intrinsically upregulated by gene-dependent characteristics causing isogenic cells within a population to lean more towards one cell fate or another, playing a part in development [27,28,29,30,31,32,33,34]. For example, work using a *Nanog* reporter line in mice showed that mESCs may have a very permissive chromatin environment for transcriptional bursting, and this behaviour may help them maintain their pluripotent state, or help decide cell fate stochastically [28].

## 3. Models of Bursting Behaviour and Their Relevance to Epidermal Differentiation

A major issue in the field is that the analysis of in vivo transcription dynamics has mainly been investigated with reporter genes and only a small number of endogenous genes. A popular system used to obtain real-time transcriptional event information is the MS2 stem–loop reporter system. This combinatory genetic/fluorescent system uses the natural interaction of stem–loop RNA structures, contained in the MS2 phage genome, with fluorescently labelled phage coat protein. Inserting phage-loop RNA at the end of a reporter gene, and co-incident expression of the tagged phage coat protein allows visualisation of single gene transcription events [8].

Single-cell RNA sequencing (scRNA-seq) can be used to probe transcriptional bursting in systems to complement the validation of these events using fluorescent reporter gene systems. In a mammalian lymphoblastoid cell line one group used a ‘pool and split’ technique of sequencing both single cells and sequencing lysed cells in pools (30 and 100 cells) of varying sizes. From this it appeared that scRNA-seq can capture some genes that are stochastically expressed due to biological reasons, not just technical noise [35]. Monoallelic expression and heavily biased autosomal expression of one allele was observed, one explanation of which is bursting—other explanations are technical noise, or stochastic events due to low single molecule capture efficiency.

In order to understand the experimental data produced by these experiments, gene bursting in eukaryotes has been modelled in various different ways. The use of two or more states to reflect the different conditions any gene can be in, such as varying degrees of activity, is central to this modelling. In two-state models, a gene can be either in an ‘on’ or ‘off’ state, one of the most notable being Peccoud and Ycart’s two-state telegraph model, which posits that a promoters state is controlled by intrinsic variability in transcription initiation [36]. The two-state telegraph model has three parameters—the burst duration, size, and frequency—with ‘size’ being the number of RNA polymerases loaded onto the gene. This has been seen to be an oversimplification in many systems, with key evidence against this model being the presence of a refractory period in gene expression in mammalian cells [37,38,39]. Other models of transcriptional bursting have been suggested that allow for more complex kinetic behaviour and more points of control [40,41]. The number of rate-limiting steps (states the promoter can be in) also tunes noise levels as much as burst size and frequency [42].

Transcriptional bursting has additionally been linked to the resource limitations cells experience, and the way they share resources for processes [43]. Terminally differentiating keratinocytes experience these kinds of stressors constitutively due to moving away from the resource supply of the vasculature in the dermis. If keratinocytes can “sense” the environment this may be a trigger for increased transcription and nuclear degradation occur in the upper epidermis.

## 4. Modulators of Bursting Behaviour and Their Links to Epidermal Differentiation

As mentioned above, bursts can be defined by several parameters including their duration, size and frequency. Another metric that is also being used is ‘burst fraction’, the proportion of time each transcription site transcribes the gene within a cell population [44,45]. Attempts at establishing how cells control the parameters of transcriptional bursting have been attempted in many model systems, cell lines and tissues [46,47,48]. Gene dosage during the cell cycle is modulated by burst frequency [49], which suggests that bursting could also be a method of controlling changes in transcription we and others have seen in late epidermal terminal differentiation using scRNA-seq ([48,49,50,51], Figure 1).

In *Dictyostelium discoideum* MS2 reporter genes revealed that the promoter is the dominant factor for determining bursting behaviour [52]. However, perturbing the TATA box only altered the number of initiation events, and did not affect the duration or frequency of active states [46]. Work in *Drosophila* shows two gene promoters can synchronise bursting when activated by the same enhancer [53], suggesting that enhancers may also have a role in controlling burst timing. It was found that strong enhancers drive higher frequency bursts than weak enhancers. Super enhancers seemed to show a relatively constant level of transcriptional activity. A model developed later [54] showed that enhancers display this behaviour seen in the quantitative imaging data from Fukaya et al. Bursting continued until phase separation from the surrounding molecules of the binding complexes (transcriptional activity saturates) was sustained, whereafter burst fluctuations reduced [54].

In mouse fibroblast cells, longer gene loci have smaller-sized bursts and certain promoter elements, such as a TATA box, have a dominant effect on burst size [55]. The same study also found enhancer polymorphisms affect burst frequency, but not size. Other studies have also found that cis-regulatory elements have a markedly large effect on the expression of genes in mammalian cells [39]. Further work showed that small individual elements of the promoter could modulate individual bursting parameters [56]. Therefore, the genomic environment modulates the bursting behaviour.

Reporter genes inserted into the genome will show bursting, whilst the same reporter on a plasmid does not [57]. Randomly integrating reporter genes into the genome results in different burst size and frequency, providing further evidence that chromatin environment may be a determinant of behaviour [58]. However, this also may be influenced by cis-acting factors such as enhancers [58]. Histone acetylation of reporter genes influenced the frequency of transcriptional bursts in mouse fibroblast cells, indicating accessibility of chromatin is a key factor in determining bursting behaviour in mammalian cells [59].

Another study has shown that raising the number of chromatin contacts on the β-globin gene promoter increases the burst fraction, but not the burst size during erythroid maturation if you force those contacts [44]. Furthermore, enhancer deletion reduced both burst size and fraction compared to normal maturation. In mouse neurons, modulating histone acetylation on enhancers also caused correlated modulation of burst dynamics [60]. scSLAM-seq, a variant of scRNA-seq, used to investigate transcriptional dynamics in mouse fibroblast cells infected by a cytomegalovirus, demonstrated that gene-specific effects were more important than cell-specific effects for determining bursting behaviour [61]. This evidence taken together shows the modulation of bursting behaviour in mammalian cells is complex and only just beginning to be elucidated. Gene-specific effects, particularly those relating to the promoter, appear to have a great effect on the behaviour, as well as chromatin state. This suggests that the kinetic behaviour of early transcriptional activators may be the initial cause of transcriptional bursts, while the duration and size of the bursts may be modulated by other factors.

Transcription factors (TFs) are essential to the assembly of the basal transcription machinery on gene promoters. Therefore, TFs may be one of the earlier points by which bursting behaviour is regulated dynamically. Imaging has provided evidence that local transacting factors at the gene promoter can affect the frequency but not the size of bursts [62]. However, in Drosophila embryos TFs control the rate of transcription by altering burst frequency [53,63]. Work using *Neurospora* involved the development of simple models studying the kinetics of bursting in relation to TF binding [64]. It found that TFs may only need to interact with target genes transiently to activate them, which questions the importance of TFs assembling the rest of the basal transcription machinery given their transient interaction. Instead, TFs may impart a ‘memory’ on the bound chromatin activating or repressing the gene. Work using the glucocorticoid receptor found TFs mobility affects burst duration, while the bound fraction to the promoter affects the size [65].

External factors, such as intercellular signals can also have an effect on the measurable parameters of transcriptional bursting. In vitro, culture conditions such as inhibiting signalling pathways can result in changes in transcriptional burst size and frequency. The stochastic expression of Nanog in mouse embryonic stem cells was affected when changing between serum and inhibited conditions [66] and consistent with this, serum-induced expression of actin augments both burst frequency and burst size, while serum induction of c-Fos leads to an increase in burst frequency without changing burst size [67,68]. The activation of B cells by interferon-γ also resulted in differing bursting behaviour compared to the constitutive transcription shown in unactivated B cells [56]. This adaptability to stimulus may indicate bursts of transcription may aid cells responding to stimuli. Alternatively, the fact transcription is stochastic may in itself allow rapid responses [64].

A variety of signalling pathways have been shown to influence bursting behaviour. Natural cAMP concentration fluctuations oscillate with the same period as transcriptional bursting activity in *Dictyostelium* [69]. Endocrine signalling using TGF-β1 [70], the glucocorticoid receptor [65], and transcription factor concentration [68] in mammalian cells both influenced the bursting behaviour of selected genes. Intrinsic properties of individual cells such as cell volume and cell cycle stage have also been shown to influence bursting activity [49,71]. Keratinocytes undergo several signals such as calcium signalling [72,73] as well as becoming large and flattened during terminal differentiation. Could these be the cue for eliciting increased bursting and transcription in the epidermis?

## 5. Stochastic Transcription in Mammalian Cells

Mammalian transcription appears to be dominated by bursting. Experiments with lentiviral vectors in mammalian cells analysing 8000 different genomic loci found that episodic bursts of transcription occurred at almost all of them [58]. However, as mentioned above, a notable difference observed in mammalian cells is that pulsatile expression appears to have a refractory period in which gene transcription cannot be initiated [37,38,39]. This refractory period can be modelled with other organisms with similar properties of transcriptional behaviour, such as *Neurospora*, and has been shown to allow fast responses to stimuli [64].

Studies in mouse fibroblast cell lines found burst frequency and size are uncoupled, indicating they have differing molecular controls [59]. A transcriptional bursting model closely correlates with observed imaging data in tissue sections of mouse kidney for monoallelic expression of genes, which further links protein translation levels to the level of bursting [74]. In some systems, such as neural development, there is a prolonged “on” state in which a neuronal genes expression is upregulated by increased bursting events [75].

Transcriptional noise in the mouse embryo is highest before cells become committed to a differentiation pathway, and subsides after commitment [31]. Therefore, stochasticity in the early mouse embryo has been postulated as a model for cell-fate decisions in early mammalian development [76]. High noise may make cells developmentally competent, allowing them to be more flexible in their response to cues to develop/differentiate. This mechanism has been postulated for factors such as Nanog, which is stochastically regulated, allowing mESCs to explore pluripotency [28]. Increased noise in other systems, such as erythroid progenitors is observed [77], before irreversible commitment to cell fate [78]. Transcriptional stochasticity could translate into various aspects of keratinocyte differentiation, such as the exit of the cell cycle in proliferating basal keratinocytes, or in later stages of cornification.

In a similar fashion to keratinocytes, lens epithelial cells have a program of differentiation that requires the removal of all organelles, coincident with high expression of crystallin genes required for lens transparency. Possible sites of crystallin genes are ‘protected’ from degradation until the last possible moment [45]. In these cells, the burst fraction—the proportion of time each transcription site transcribes the gene within a cell population—was highly variable, while the intensity of the burst, i.e., the level of transcription, was roughly constant. This may mean that when mRNA synthesis has to be increased, as with lens cell differentiation, the burst fraction parameter is modulated, rather than the intensity. Further research in lens fibre cells focusing on bidirectional promoters has shown that bursting behaviour of crystallin genes can be synchronized due to proximal genomic positioning of the genes promoters [79]. Another inference that can be made from both studies is that nuclei are transcriptionally competent right up until destruction. Lens fibre cells have been compared to keratinocytes due to their analogous enucleation processes [80]. This may indicate that keratinocytes are also transcriptionally competent up until enucleation, or even further into cornification.

A model used to control for factors such as cell size and other cell state features showed, however, that the contribution of transcriptional bursting may be over exaggerated in many studies [81]. The model indicated that cells still showing stochastic expression here had variability close to the Poisson limit. The gene expression observed in many studies may therefore be due to natural variability in underlying cell state [81].

The overall noise profiles of eukaryotic genes are likely due to a interplay of transcriptional and translational regulatory events [82]. Steady-state abundance of mRNAs is potentially down to largely contextual factors, not bursting, as things such as the mRNA residence time in the nucleus buffer their cytoplasmic concentration [83,84]. This means the nuclear membrane serves to ‘split the steps of the central dogma’ [84] to prevent transcriptional noise from impacting protein production at polysomes. However, new techniques, have allowed investigations of single-molecule translation analogous to the MS2-phage system for RNA [85], revealing translation also shows bursting behaviour. It has been implied the nucleus is like a ‘bucket’ [84], stockpiling transcribed RNAs due to the slow speed of RNA transport out of the nuclear pore complex (NPC). This nuclear retention may lead to bursts of translational activity as transcripts are exported to the polysomes. However, scRNA-seq approaches likely distort the abundance of transcripts. Therefore, the findings made by Battich and colleagues may mean protein bursting, such as transcription, is due to cells lacking contextual factors rather than solely transcriptional bursting [58].

## 6. Biomolecular Condensates and Their Potential Roles in the Epidermis

Recent data have shown that membraneless bodies which behave like phase-separating bodies can be formed from filaggrin repeats in granular layer keratinocytes [86]. Ablation of these bodies results in reduced epidermal barrier function, suggesting they have a key role in proper epidermal development. Our proposal is that these bodies are a form of biomolecular condensate and could be related to other bodies we have observed in granular keratinocytes group ([5], Figure 1).

The term biomolecular condensates (BMCs) is used to describe subcellular compartments within eukaryotic cells which are non-membrane bound [87]. They are at the micron scale and are involved in hugely diverse processes within the cell, such as ribosome biogenesis and RNA metabolism [88,89]. Many experiments have looked at the physical properties of how condensates form, mainly in vitro. BMCs can exchange their constituents with the surrounding solvent, which has been shown using live cell imaging and photobleaching experiments with nuclear BMCs [90,91,92]. They also exhibit behaviour such as fusion and fission events between like condensates [93,94,95,96,97,98].

BMCs are formed by cooperative interaction between polynucleotides and proteins. Mixtures of RNA and RNA-binding proteins form these condensates in vivo [99,100,101,102,103]. High concentrations of proteins containing intrinsically disordered regions (IDRs) will also form condensates via phase separation in vitro [104,105,106,107]. Mutations in these IDRs can even impair the function of the BMCs that they are components of [108,109].

The internal structures of BMCs are not homogenous. For example, co-existing subcompartments within the nucleolus have been identified. Multilayered liquid droplets may facilitate sequential reactions with differing conditions, such as rRNA processing in the nucleolus. These subcompartments form droplets in vitro when components are isolated, and will even form a multiphase droplet when recombined, recapitulating an approximation of their in vivo conditions [100]. Additionally, these condensates have even been shown to have distinct phases of assembly in vivo [102].

BMCs do not use lipid-based membranes to separate themselves from their surrounding media, and instead a model based on liquid–liquid phase separation (LLPS) has been developed to understand their behaviour and dynamics [87]. However, these models have only been tested in in vitro and in silico and are yet to be elucidated in vivo [110]. There are other possible models used to explain condensate behaviour, but for the purposes of this review we will assume LLPS is the accepted model.

Two cytosolic examples of these BMCs are P bodies (PBs) and stress granules (SGs) which are RNP granules that typically form in the cytosol as a consequence of altered RNA homeostasis [111,112,113]. P bodies contain mRNAs which are associated with repressive molecules for translation, as well as machinery for mRNA decay. mRNAs within P bodies can be de-capped and digested, but RNA degradation does not solely occur in P bodies [114]. Stress granules form from mRNAs which are stalled in translation initiation and contain translation initiation factors, RNA-binding proteins (RBPs), and many other non-RNA binding proteins [115]. They are sites of storage and triage for mRNAs in times of cell stress. In fact, studies have found that 50% of poly(A)+ RNA in mammalian cells can be found associated to SGs under stress [116]. Polysome disassembly, caused by a reduction in the pool of initiator ternary complexes required to begin protein synthesis during stress, is rapidly followed by SG formation as the RNAs are routed to SG formation sites [117]. Interestingly, protein transcripts synthesised in response to stress may be selectively excluded from SGs. mRNA coding for HSP70, a ‘disaggregase’ protein synthesised by cells under stress, is selectively excluded from SGs [117].

SGs and PBs are considered distinct organelles. However, a percentage of them within cells are found docked against each other [118,119]. Given their function, this suggests a dynamic cyclical process where mRNPs can be remodelled and exchanged between these structures. The connected state of these curious organelles has been further demonstrated between cell compartments. The formation of paraspeckles, a nuclear RNP, has been shown to be regulated by SGs in the cytoplasm, indicating the existence of an ‘RNP granule continuum’ within cells [120]. Exchange of constituents between these granules, and cellular compartments may be vital for a cell’s physiological response to stress.

Mutating or deleting BMC component molecules will reduce granule formation in vivo [112,121]. For example, *Saccharomyces cerevisiae* with an *Edc3p* deletion show a strong reduction in the number of P bodies observed [122], and mammalian cells require Ras-GAP SH3 domain binding protein (G3BP) for SG formation during oxidative stress [123]. These proteins all bind RNA and could help assemble P bodies into larger structures via protein-protein interactions.

## 7. Are Condensates and Transcription Linked?

PBs and SGs are known to form due to altered RNA homeostasis [111,112,113]. Given that keratinocytes are undergoing transcriptional changes during terminal differentiation, we posit that the observed bodies [86] may be a consequence, or a functional part, of the translational response in terminal differentiation.

Housekeeping genes are generally sequestered in RNPgs. This could be a way of freeing up polysomes for massive translational upregulation of tissue-specific genes. This could allow the setting up of expression gradients in development. In keratinocytes, this could be a way of prioritising the genes required during epidermal terminal differentiation. Tissue- or cell-specific RNPgs have been identified, with Balbiani bodies and germ granules being exclusively found in germ cells, and RNA-transport granules exclusive to neuronal cells. The BMCs observed by Quiroz et al. [86] may be an epidermal-specific form of BMCs as filaggrin expression is restricted to this tissue and involved in terminal differentiation.

It should be considered that the phospho-Lamin A-positive cytoplasmic RNP granules are likely to be symptomatic of higher expression but may not regulate it. This may primarily have an effect on translation, Therefore the presence of these granules could affect the proteome, rather than the transcriptome, by controlling protein concentration noise [124].

## 8. Evidence for Transcriptional Bursting and Ribonuclear Protein Granules in Epidermal Terminal Differentiation

The similarities between how lens epithelial cells and the keratinocytes differentiate would suggest that there is a clear role for RNPgs and pulsatile expression of genes. How do these phenomena intersect with processes occurring during epidermal differentiation, in particular the destruction of the nucleus?

While the field of transcriptional bursting has been rapidly adding to the evidence for the phenomena there are some good examples that indicate that while transcription may occur in bursts, protein abundance resulting from this mRNA bursting is negligibly affected [83,84]. As nuclear export of mRNA is slow compared to transcription, this rate-limiting step effectively acts as a buffer for transcript abundance in polysomes. However, this then presents an interesting idea in keratinocytes as their nuclear lamins are phosphorylated and degraded during nuclear destruction [4,5]. If transcription continues beyond this point, there will be no buffering provided by the nuclear lamina, and protein abundance could be affected also. This has been seen in studies of systems which had fewer contextual factors to buffer protein expression [58].

RNP granules appear as spherical objects, often forming a ‘corona’ where multiple key components are formed, with distinct layers forming the outer shell and inner core [103]. Imaging of serine 404-phosphorylated-lamin A (pLMNA) in terminally differentiating rat keratinocytes has shown the dispersal of spherical ‘bodies’ around the perinuclear area and throughout the cytosol of these cells (Figure 1B). Lamin A has many binding partners, and significant parts of it are intrinsically disordered [125,126]. Therefore, it could form the scaffolds for phase-separating species which form the observed objects. We hypothesise that these could be RNP granules involved in the process of terminal differentiation, and that pLMNA could be a scaffold protein for their formation [87].

Additionally, there are other lines of evidence phase-separating condensates may play a role in keratinocyte terminal differentiation. Actin is actively remodelled during the epidermal terminal differentiation process. AKT1-phosphorlyated HSP27 stops stabilising cortical actin leading to collapse of the network concomitant with nuclear destruction [127]. The ARP2/3 complex has been shown to be involved in normal epidermal differentiation of keratinocytes [5,128]. Additionally, experiments have shown that binding partners of the ARP2/3 complex undergo phase transition [103], suggesting that actin could be a key contributor to condensate formation in terminally differentiating keratinocytes.

Keratinocytes are under a number of different stresses in the final stages of term differentiation: modified apoptosis, autophagic destruction of the nucleus and organelles [129], oxidative stress [130], as well as the unfolded protein response (UPR) and increase in calcium concentration in the upper epidermis [72]. The ER is closely related to the UPR and ER stress has been shown to activate autophagy in keratinocytes, a process linked to the destruction of keratinocyte organelles [129,131]. This stress may promote the formation of membraneless organelles such as SGs in differentiating keratinocytes, or novel BMCs of similar composition.

Stress granules have been proposed to modulate some pathways that are key to keratinocyte terminal differentiation such as the mTOR, as well as RACK1 and TRAF2 signalling pathways. They may modulate these pathways by sequestering components of the pathways into a condensate [132,133,134,135]. RACK1 modulates many pathways involved in epidermal homeostasis such as the PKC/MAPK signaling pathways [136]. The TRAF2-caspase pathway modulates caspase-8, which, while not being linked to keratinocyte differentiation directly, has been linked to inflammatory skin disease [137]. The mTOR signaling pathway is known to modulate epidermal development [138], and the mTORC2 pathway is critical to epidermal terminal differentiation and barrier function [138,139,140,141], in part by modulating LMNA degradation and nuclear degradation processes [4]. This suggests that stress granules, or a form of them, are involved in keratinocyte terminal differentiation. Isoforms of stress granules may contain novel components such as pLMNA, or exist alongside the observed pLMNA bodies.

Another approach to understanding a possible role of ribonuclear granules and transcriptional bursting in epidermal differentiation and nuclear destruction is looking at diseases that have impaired terminal differentiation and nuclear degradation, such as psoriasis and atopic dermatitis that are common skin diseases that display hyperkeratosis (impaired stratification) and parakeratosis (nuclei retained in the granular layer of the epidermis). Coiled-Coil alpha-helical Rod protein 1 (CCHCR1) is a component of P bodies [142], and in the *PSORS1* locus, a risk locus for psoriasis, could modulate the localisation and function of P bodies in patient keratinocytes that were positive for the SNPs associated with *PSORS1* [143].

Additionally, lens epithelium-derived growth factor/dense fine speckles 70 kDa protein LEDGF/DFS70, is a major autoantigen in atopic dermatitis, with 30% of patients having antibodies to this protein. LEDGF activates expression of crystallins in lens epithelial cells [144,145]. LEDGF expression increases with ER stress and is increased in expression in the upper epidermis specifically in keratohyalin granules, co-localising with filaggrin in these granules.

Eukaryotic transcription appears to be dominated by stochastic bursts, suggesting that genes involved in keratinocyte terminal differentiation could be transcribed stochastically. Transcriptional events could occur stochastically within the terminally differentiating population of granular keratinocytes, much like other documented cell types, which explore differentiation states before commitment [28,77,78]. Genes from the EDC are activated co-ordinately in epidermal terminal differentiation during development of the embryo [146], which can be used as a proxy for mature developing epidermis. This, along with other scRNA-seq studies [147], indicate that a large burst of transcription occurs in terminal differentiation. To elucidate whether this transcription is stochastic, or if it follows a clear progression of sequentially expressed genes, will require further research.

Transcription in terminally differentiating keratinocytes could potentially continue beyond nuclear permeation, meaning these bursts would be unbuffered by the nuclear envelope and export mechanisms involved in normal RNA transit. This would lead to rapid concentration spikes of mRNA in the cytosol, unbuffered by NPC export, potentially allowing local concentrations of RNA and protein to phase separate, which could form granules. We show RNA localising to bodies in the cytosol of granular keratinocytes concomitant with lamina degradation (Figure 1). These bodies could be RNPgs.

**Figure 2:**
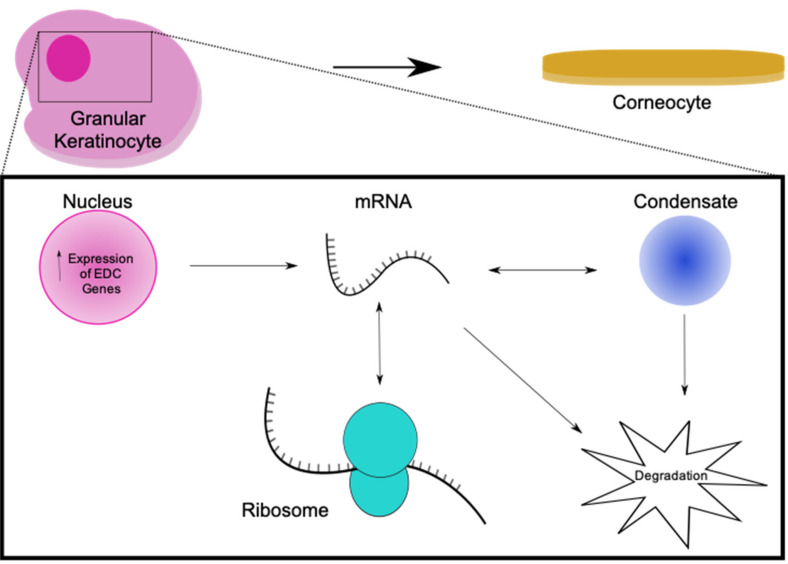
The potential role of condensates in keratinocyte terminal differentiation. In a granular keratinocyte undergoing the last stages of terminal differentiation, condensates may form from the excess RNA being produced by loci such as the EDC. These condensates may act as a sink for mRNA, either targeting it for degradation or allowing it to re-enter the translational pool from the condensate.

These putative RNP granules could form a sink for mRNAs being transcribed (Figure 2), before or after permeation, protecting them and allowing them to be translated during this process as the cornified envelope is formed. This process would likely have parallels with the ‘mRNA cycle’ proposed for P bodies harbouring mRNAs which can be degraded or re-enter the translational pool [148,149]. Some BMCs are now being isolated effectively, allowing study of their molecular components and behaviour in vitro [150]. Characterisation of their ‘proteome’ and ‘transcriptome’ will allow functional insight. The question remains of whether these RNP granules are an artefact of the cell death-like process of cornification, or whether they have a functional role in normal terminal differentiation.
